# The Marine Compound Tartrolon E Targets the Asexual and Early Sexual Stages of *Cryptosporidium parvum*

**DOI:** 10.3390/microorganisms10112260

**Published:** 2022-11-15

**Authors:** Alexis Cotto-Rosario, Emma Y. D. Miller, Fernanda G. Fumuso, Jason A. Clement, Matthew J. Todd, Roberta M. O’Connor

**Affiliations:** 1 Department of Veterinary Microbiology and Pathology, Washington State University, Pullman, WA 99164, USA; 2 Department of Veterinary and Biomedical Sciences, University of Minnesota, St. Paul, MN 55108, USA; 3 Baruch S. Blumberg Institute, Doylestown, PA 18902, USA

**Keywords:** antiparasitic compound, cryptosporidiosis, *Cryptosporidium*, drug candidates, life cycle stages, marine natural products, phenotypic assays

## Abstract

New therapeutic agents for cryptosporidiosis are a critical medical need. The marine organic compound, tartrolon E (trtE), is highly effective against multiple apicomplexan parasites, including *Cryptosporidium*. Understanding the mechanism of action of trtE is required to advance in the drug development pipeline. Here, we validate using Nluc *C. parvum* parasites for the study of trtE and pinpoint the life stage targeted by trtE. Results show that trtE kills Nluc and wild type *C. parvum* with equal efficiency, confirming the use of the Nluc *C. parvum* to study this compound. Results revealed that trtE kills the parasite within an hour of treatment and while the compound has no effect on viability of sporozoites, trtE does inhibit establishment of infection. Targeting treatment at particular life cycle stages demonstrated that trtE is effective against asexual of the parasite but has reduced efficacy against mature sexual stages. Gene expression analysis shows that trtE inhibits the early sexual stage of the parasite. Results from these studies will aid the development of trtE as a therapeutic for cryptosporidiosis.

## 1. Introduction

Cryptosporidiosis is a diarrheal disease that affects 2 out of 100,000 people yearly in the United States [1]. This infection is acquired by ingesting food or water contaminated with oocysts of the protozoan parasite *Cryptosporidium* [2]. Cryptosporidiosis is recognized worldwide as a leading diarrheal illness in children under five years old [3,4,5] and in the immunocompromised population, such as individuals with AIDS and organ transplant patients [6,7]. Immunocompromised patients suffer from chronic episodes of fulminant diarrhea that lead to severe dehydration, secondary gastrointestinal disorders, and, in extreme cases, death [8]. Even healthy adults that suffered from cryptosporidiosis report underlying symptoms five years after the initial infection event [9].

Once ingested, *Cryptosporidium* oocysts excyst in the intestine, each releasing four sporozoites that invade enterocytes. The parasite replicates within a unique parasitophorous vacuole that is intracellular but extracytoplasmic [10]. The genesis of the parasitophorous vacuole and development into the trophozoite stage occurs rapidly after invasion of the host cell [11]. Trophozoites divide asexually and develop into meronts at 10–12 h post-infection (hpi), after which merozoites egress to infect new host cells [12]. *Cryptosporidium* cycles through merogony three times before male and female gametes start to appear at 36–42 hpi, allowing *Cryptosporidium* to reproduce sexually within its host [12,13]. Fertilization leads to the development of two types of oocysts; thin-walled oocysts that excyst within the host leading to autoinfection, and thick-walled oocysts that are shed in the feces into the environment [10].

Current treatment for cryptosporidiosis relies on nitazoxanide, the only FDA-approved drug for this disease. Recently, there have been reports of reduced efficacy and resistance to nitazoxanide [14]. Furthermore, nitazoxanide has proven ineffective in immunocompromised individuals [14,15]. Thus, there is a need for novel therapeutics to be identified, characterized, and introduced into the drug development pipeline.

Tartrolon E (trtE), a polyketide natural product isolated from a shipworm symbiotic bacterium [16], inhibits several apicomplexan parasites at nanomolar concentrations in vitro, including *Cryptosporidium parvum* [17]. Furthermore, trtE is highly effective against *C. parvum* in neonatal mice [17], supporting further investigation of this compound as a potential therapeutic. The target of trtE has not been identified [17,18] therefore it is critical to identify the specific life cycle stages and processes that the compound targets. This data will inform development of trtE therapeutic strategies, aid in choosing appropriate partner compounds to prevent resistance, and advance progress of trtE through the therapeutic pipeline. 

## 2. Materials and Methods

### 2.1. Cell Maintenance and Parasite Strains

Iowa strain transgenic *C. parvum* oocysts expressing nanoluciferase (Nluc) [19], as well as wild-type (WT) *C. parvum* oocysts, were acquired from the University of Arizona in Tucson, AZ (https://acbs.arizona.edu/cryptosporidium-production-laboratory (accessed 23 on October 2022)). Human ileocecal colorectal adenocarcinoma cells were obtained from ATCC (HCT-8, ATCC^®^ CCL244^™^) and maintained as recommended. 

### 2.2. Compounds

*Teredinibacter turnerae* T7901 was cultured and trtE purified from the bacterium as described [17], with some modifications. From a 12-L *T. turnerae* fermentation batch, the broths were centrifuged at 16,264× *g* for 20 min. A total of 1.2 L of acetone was added to the cell pellets, and the mixtures were shaken for 1 h on an Eberbach shaker at room temperature. The acetone extract was filtered (Whatman™ grade 1) twice to remove most of the cell debris. The pooled acetone extracts were then concentrated to dryness by rotary evaporation. The acetone extract (880 mg) was suspended in about 10 mL DMSO and centrifuged. The DMSO-soluble material was charged to a 43 g CombiFlash RediSep^®^ Rf column. The insoluble material was suspended in 50% aqueous acetone and applied to the column. The column was eluted with a solvent gradient: (A = water, B = acetonitrile) 50% B for 2 min.; 50% B to 100% B, 20 min.; hold at 100% B, 10 min. The solvent flow rate was 40 mL/min. Four fractions were collected between 0–12.5 min., and 15 mL fractions were collected beginning at 12.5 min. Fractions 18–52, which contained trtE, were combined and dried by rotary evaporation. The pool of fractions from the flash C18 column separation was dissolved in 4 mL of chloroform and applied to a 12 g CombiFlash Gold^®^ silica gel column pre-equilibrated with hexanes. The silica gel column was eluted with a solvent gradient: (A = hexanes, B = ethyl acetate) 0.5 min, 0% B; 0% B to 5% B, 10 min.; 5% B to 25% B, 10 min.; hold at 25% B 6 min. The solvent flow rate was 30 mL/min. Eighty-one fractions were collected. Fractions 58–66 were pooled and concentrated under vacuum to afford 70 mg of trtE. All batches of trtE were verified by mass spectrometry and NMR (Supplementary Figure S1). MMV665917 was provided by Dr. Christopher Huston (University of Vermont, Burlington, VT, USA). Wiskostatin was purchased from Millipore Sigma (Catalog #W2270). All compounds were diluted in 100% DMSO. Aliquots were stored at −80 °C and subjected to no more than five freeze–thaw cycles.

### 2.3. In Vitro Growth Inhibition Assay

We have previously described this assay in detail [20]. Briefly, HCT-8 cells were seeded in 96-well, white sided, clear bottom plates and allowed to grow to confluency. WT and Nluc *C. parvum* oocysts were treated with 10% bleach and then washed three times with 1X PBS. Oocysts were then resuspended in complete media with 0.6% taurocholate acid (Santa Cruz Biotechnology, Santa Cruz, CA, USA) and introduced to the cell monolayer at 10,000 oocysts per well. The plate was centrifuged at 1000× *g* for 5 min to maximize sporozoite contact with host cells. Infected cells were treated at different times post infection, and for varying durations, with trtE. DMSO was run in parallel as a vehicle control. Parasite growth was measured either by relative luminescence (Nluc *C. parvum*) or qPCR (Nluc and WT *C. parvum*) at 72 hpi. Relative luminescence units (RLU) were measured using a SpectraMax^®^ L Microplate Reader (Molecular Devices, San Jose, CA, USA). Percent inhibition of Nluc parasites by trtE was calculated relative to the DMSO control with the formula: % Inhibition = [(RLU_DMSO_ − RLU_compound_)/RLU_DMSO_] × 100.

### 2.4. Quantification of Parasite Growth by qPCR

DNA of cells infected with Nluc and WT parasites was isolated using the Wizard^®^ gDNA isolation kit (Promega, WI, USA) following the manufacturer’s protocol. DNA amplification was quantified in an Mx3500P qPCR system with MxPro Software (v.4.10) (Agilent, Santa Clara, CA, USA) using SYBR^®^ green Supermix (Bio-Rad Laboratories, Hercules, CA, USA). To quantify parasite growth, *C. parvum* gp40/15 gene (*Cp_gp40/15*) primers were used [21]. Human actin primers were used to amplify the reference gene [22]. Relative abundance of the *C. parvum* gene was calculated using the 2^−∆∆Ct^ method [23]. Percent inhibition of parasite growth by trtE was calculated relative to DMSO treated controls.

### 2.5. Determination of Oocyst Excystation Rate

Oocysts were bleached, washed, and counted on a hemocytometer. The oocysts were then divided into two aliquots and either 100 nM trtE or DMSO (vehicle control) were added to the oocysts. Oocysts were then incubated for 2 h in a 37 °C water bath at which time the excystation rate of each sample was determined by counting the remaining non-excysted oocysts. Percent excystation was calculated using the formula: % excystation = ((intact oocysts_before tmt_ − intact oocysts_after tmt_)/intact oocysts_before tmt_)) × 100.

### 2.6. Sporozoite Viability Assay

*C. parvum* sporozoites were tested for viability by modifying a previously described assay [24]. Briefly, 5 × 10^6^ oocysts were bleached, washed with 1X PBS and the oocysts suspended in 0.6% taurocholate acid in cell culture medium. Oocysts were excysted at 37 °C for 30 min with mixing every 5 min. Excysted oocysts and sporozoites were aliquoted and treated with either 100 nM trtE or DMSO for 1 h at 37 °C. For a positive control for killing, sporozoites were either treated with 10% formaldehyde and heat shocked for 2 min at 60 °C or heat shocked only. To label viable parasites, treated sporozoites were then incubated with CFSE a final concentration of 10 µM (Invitrogen, C1157) at 37 °C for 30 min. DNA was labeled with 0.9 mM Hoechst (Abcam, ab1455971). Samples were evaluated by fluorescence microscopy (Zeiss^®^ Axioscope 5). Slides were blinded and the number of viable sporozoites per 200 sporozoites were counted for each sample. The Jenoptik GRYPHAX^®^ camera and its image acquisition software (v2.2.0) were used to take representative images of sporozoites.

### 2.7. Invasion Inhibition Assay

This method was modified from an assay described previously [11]. Briefly, HCT-8 cells were seeded on cell culture chamber glass slides (NEST^®^ Scientific, Woodbridge, NJ, USA) until confluent and treated for 1 h with either trtE, DMSO, or wiskostatin. Wiskostatin was reported to inhibit *C. parvum* invasion [11] and is used here as a control. HCT-8 monolayers were then infected with 50,000 NLuc oocysts per well. Heat inactivated oocysts were used as negative control. After 3 h of incubation, cells were washed three times with 111 mM D-galactose in 1X PBS, fixed with 4% paraformaldehyde, and permeabilized with 0.25% Triton-100X. Staining of parasitophorous vacuoles was performed with 1.33 µg/mL of fluorescein-label *Vicia villosa* lectin (VVL) (Vector Laboratories, Newark, CA, USA). Nuclei staining was performed with 0.09 mM Hoechst. Slides were blinded and stained vacuoles from at least 10 randomly selected fields were counted in each sample at 40X magnification (Zeiss^®^ Axioscope 5). Percent inhibition was normalized to DMSO as described above. The Jenoptik GRYPHAX^®^ camera and its image acquisition software (v2.2.0) were used to take representative images of infected monolayers.

### 2.8. Quantification of Gene Expression by RT-qPCR

Total RNA of *C. parvum*-infected HCT-8 cells was isolated using the RNeasy^®^ Mini Kit (QIAGEN, Hilden, Germany). Synthesis of cDNA was performed using the SuperScript™ III First-Strand Synthesis System (ThermoFisher Scientific, Waltham, MA, USA). DNA amplification was quantified in a Mx3500P qPCR system with MxPro Software (v.4.10) (Agilent, Santa Clara, CA, USA) using SYBR^®^ green Supermix (Bio-Rad Laboratories, Hercules, CA, USA). To evaluate transition into sexual stages, primers for the *DMC1* gene were used [11]. Primers for the male gamete specific *HAP2* gene (*cgd8_2220*) (Fwd—5′-CTGGTTGGTAGGAAATGC-3′; Rev—5′-CAATATCCCCACTATTCTTA-3′) and the oocyst wall protein 4 gene (*COWP4*) [25] were used to measure microgamete and macrogamete stages, respectively. Human actin primers were used to amplify the reference gene [22]. Relative gene expression was calculated using the 2^−∆∆Ct^ method [23] and percent inhibition was calculated relative to DMSO control as previously described.

### 2.9. Statistics

Data analyses were performed using GraphPad Prism v9.2.0 (La Jolla, CA, USA). Inhibition curves were extrapolated using the log[inhibitor] vs. response-variable slope (four parameter) regression equation and compared using the extra sum of squares F test. Significant differences between experimental groups were calculated using the two-tailed unpaired *t*-test, the one-way ANOVA with Dunnet’s multiple comparison test, or the two-way ANOVA with two-stage linear step-up procedure of Benjamini, Krieger and Yekutieli for multiple comparisons correction as appropriate. The statistical test used is indicated in the figure legends. Differences were considered significant if the p value was equal to or less than 0.05.

## 3. Results

### 3.1. The Inhibition Curves of trtE against WT and Nluc C. parvum Strains Are Not Significantly Different

**Figure 1:**
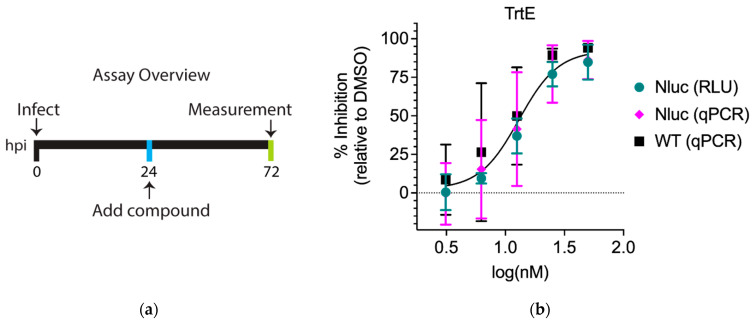
Drug-response curves of trtE against WT and Nluc *C. parvum* strains are not significantly different. (**a**) Assay overview of the in vitro growth inhibition assay in the context of a dose–response curve. (**b**) Dose–response curve of trtE against *C. parvum* strains determined with the log[inhibitor] vs. response-variable slope (four parameter) regression equation. Comparative analysis of the dose response curves using the extra sum of squares F test revealed no significant difference (*p* = 0.88). The data plotted are the means ± SD of four independent experiments.

Genetic manipulations can cause changes in sensitivity to compounds [19]. To elucidate whether there was a difference between Nluc and WT *C. parvum* strains in their susceptibility to trtE, HCT-8 cells were infected with either Nluc or WT parasites, and the infected cells treated with two-fold serial dilutions of trtE at 24 hpi. Parasite growth was quantified by nanoluciferase activity or qPCR at 72 hpi (Figure 1a). In these experiments, the effective concentration at which 50% of the parasites were inhibited (EC_50_) of trtE against Nluc *C. parvum* was 15.93 nM when determined by RLU, and 14.83 nM when determined by qPCR. The EC_50_ of trtE against WT *C. parvum* was 11.21 nM (Table 1). Comparative analysis using the extra sum of squares F test of the three dose–response curves identified no significant differences (Figure 1b). These results show that trtE is as effective against WT parasites as Nluc parasites, demonstrating that the transgenic parasites are not especially sensitive to the compound and can be used to explore the effects of trtE on *C. parvum*.

### 3.2. TrtE Kills C. parvum within the First Hour of Treatment

**Figure 2:**
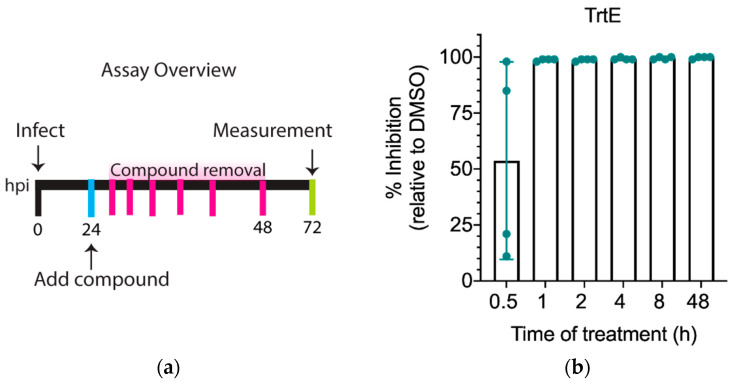
TrtE rapidly kills *Cryptosporidium.* (**a**) Overview of the inhibition growth assay when testing the time it takes a compound to kill the parasite. (**b**) Graph shows the percent inhibition of trtE treatment relative to DMSO control. The data plotted are the mean ± SD of four independent experiments.

The EC_50_ of trtE against *C. parvum* was based on a lengthy treatment period of 48 h (Figure 1a). To test the minimum time it takes trtE to inhibit parasite growth, cells infected with Nluc *C. parvum* were treated with 100 nM trtE at 24 hpi, and the compound was removed after 0.5, 1, 2, 4, 8, or 48 h of treatment. *C. parvum* growth was then measured at 72 hpi (Figure 2a). Analysis shows that parasite growth is inhibited by 99%, relative to DMSO control, after just an hour of treatment with 100 nM trtE (Figure 2b). These data demonstrate that trtE rapidly kills *C. parvum* in vitro.

### 3.3. TrtE Has No Effect on Excystation

Compounds that prevent excystation of oocysts could be employed to reduce environmental contamination or as a prophylaxis in the case of susceptible neonatal ruminants. To test whether trtE can inhibit excystation, *C. parvum* oocysts were excysted in the presence of 100 nM trtE for 2 h. The number of intact oocysts was counted before and after treatment to determine percent excystation. Excystation was not significantly influenced by trtE after 2 h of treatment when compared to DMSO (Supplementary Figure S2, *p* = 0.1503).

### 3.4. TrtE Inhibits Establishment of C. parvum Infection

**Figure 3:**
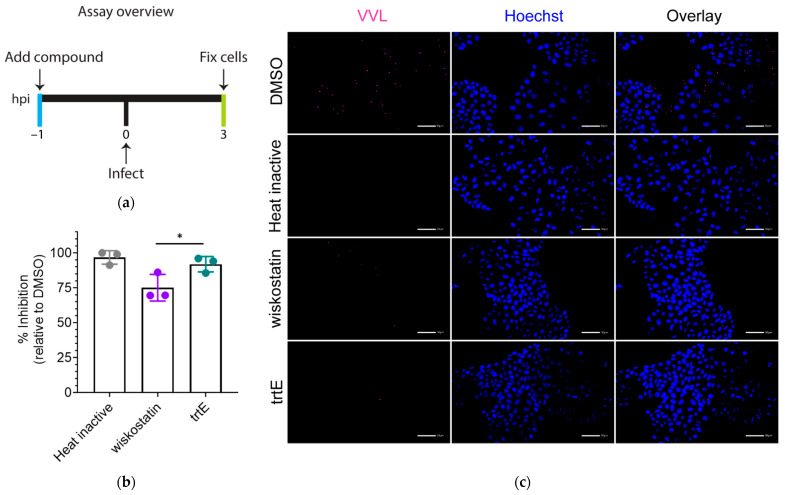
TrtE prevents establishment of *C. parvum* infection. (**a**) Overview of the invasion inhibition assay. (**b**) Quantification of parasite invasion. The graph shows percent inhibition of invasion relative to the DMSO control. Data shown are the mean ± SD of three independent experiments. Statistical significance was determined using one-way ANOVA and Dunnett’s multiple comparison test, * *p* < 0.05. (**c**) Representative images of *C. parvum* infection. Parasitophorous vacuoles were stained with 1.33 µg/mL VVL and host cell nucleus with 0.09 mM Hoechst. Scale bar represents 50 µm.

The infection assay as performed treats parasites at 24 hpi (Figure 1a), when the parasites are replicating asexually. To understand the full potential of a compound, it is necessary to identify the life stages the compound is effective against. To examine the effect of trtE on parasite invasion and establishment of infection, excysted parasites were allowed to infect host cells for 3 h in the presence of trtE or the DMSO control (Figure 3a). Parasitophorous vacuoles were stained with *Vicia villosa* lectin (VVL) and host nuclei with Hoechst (Figure 3c). Quantification of parasitophorous vacuoles shows that trtE reduces establishment of *C. parvum* infection by 90%, significantly more than the 75% reduction achieved with wiskostatin treatment (Figure 3b).

### 3.5. TrtE Does Not Reduce the Viability of C. parvum Sporozoites

**Figure 4:**
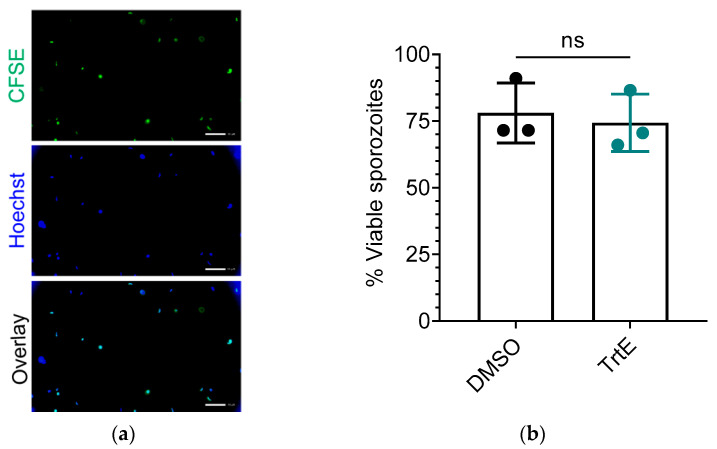
TrtE does not affect sporozoite viability. Sporozoites were labeled with 10 µM CFSE after compound treatment to determine their metabolic viability. DNA was labeled with 0.9 mM Hoechst dye. (**a**) Representative images of DMSO treated *C. parvum* sporozoites in suspension. Scale bar represents 10 µm. (**b**) Quantification of viable sporozoites. The graph shows the percent of viable sporozoites (mean ± SD of three independent experiments). Experimental groups were compared with an unpaired *t*-test; ns = not significant.

TrtE’s inhibition of early infection (Figure 3) could be the result of killing extracellular sporozoites, preventing invasion, or inhibiting early growth of the trophozoite. In a previous study we directly evaluated trtE’s effect on Toxoplasma tachyzoite infectivity [17]. However, Cryptosporidium sporozoites are fragile and damaged during centrifugation washes, preventing consistent infection. Thus, we used a viability stain (CFSE) to determine if trtE-treated sporozoites were directly killed by trtE. TrtE treated sporozoites exhibited the same viability as DMSO-treated sporozoites (Figure 4), in contrast to sporozoites killed with either 2 min heat shock (28.5% viability) or 10% formaldehyde plus heat shock (no viable sporozoites).

These data demonstrate that, since trtE has no effect on excystation nor on the extracellular sporozoite, the compound prevents establishment of infection. Since the numbers of parasitophorous vacuoles were greatly reduced in the presence of trtE, it is reasonable to hypothesize that trtE inhibits attachment and invasion mechanisms.

### 3.6. TrtE Is Most Effective during the Asexual Stage of C. parvum

**Figure 5:**
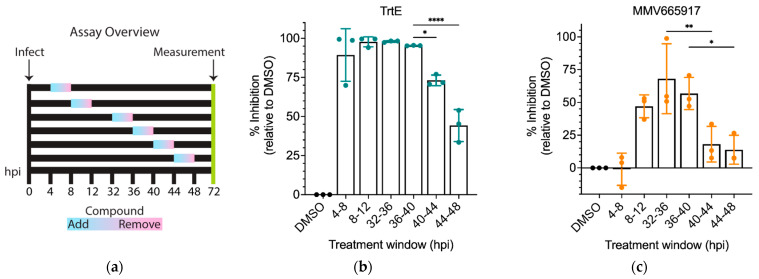
TrtE is most effective during the asexual stage of *Cryptosporidium*. (**a**) Overview of the modified infection assay to test stage specificity of the compound. Graphs show the percent inhibition of (**b**) trtE and (**c**) MMV665917 relative to DMSO. The data plotted are means ± S.D of three independent experiments. Statistical significance was determined using one-way ANOVA and Dunnett’s multiple comparison test, * *p* < 0.05; ** *p* < 0.005; **** *p* < 0.0001.

The intracellular stages present in 2D culture of *C. parvum* are the asexual stages and the microgametes and macrogametes (sexual stages). Various anti-*Cryptosporidium* compounds have been shown to target either asexual or sexual stages, or both [11,26]. A time course treatment was performed to elucidate the exact stage at which trtE inhibits the parasite (Figure 5a). Infected cells were treated with 100 nM of trtE for 4 h at 4 and 8 h, when the parasite is undergoing asexual division, 32 and 36 h when asexual division is ending and parasites are committing to sexual differentiation and at 40 and 44 hpi when the microgametes and macrogametes predominate in the culture [12]. Parasite growth was measured at 72 hpi. TrtE inhibits ≥ 90% of parasite growth during asexual replication, but the efficacy of trtE against *C. parvum* is significantly reduced at 40 to 48 hpi when gametes are present (Figure 5b). MMV665917, a piperazine that inhibits the asexual to sexual conversion of *C. parvum* [11], was run in the assay in parallel. The EC_50_ of MMV665917 was determined to find a comparable drug-response to that of trtE (Supplementary Figure S3) and it was confirmed that the compound inhibits 75% of the parasite after 4 h of treatment (Supplementary Figure S4). MMV665917, at 6 µM, does not inhibit establishment of infection (Supplementary Figure S5), fails to inhibit more than 50% of the parasites early during infection and inhibits parasite growth predominantly at 32–40 hpi, concomitant with the time that the parasite commits to gamete formation ([11] and Figure 5c). Similar to MMV665917, trtE exhibits a significant decrease in inhibition after 40 hpi (Figure 5c). These data demonstrate that while trtE is highly effective against asexual stages and MMV665917 is active during sexual differentiation, both compounds have reduced efficacy once the parasites pass the sexual stage commitment threshold.

### 3.7. TrtE Targets Early Sexual Stages of Cryptosporidium

**Figure 6:**
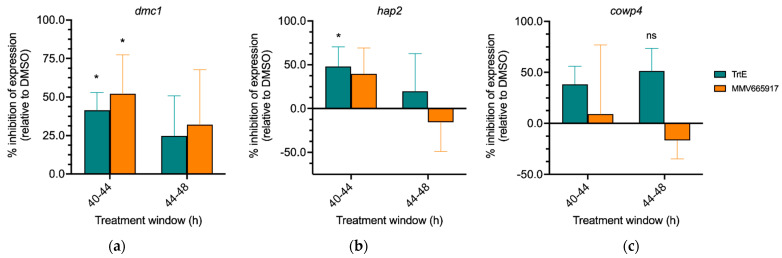
TrtE targets early sexual stages of *Cryptosporidium*. *C. parvum* infected cells were treated with DMSO, trtE or MMV665917 for 4 h at 40, and 44 hpi. RNA was isolated and RT-qPCR was used to quantify the relative expression of (**a**) *DMC1*, (**b**) *HAP2* and (**c**) *COWP4*. The human actin gene was used as the reference gene. Data plotted are the mean percent inhibition of expression relative to DMSO control based on 2^–∆∆Ct^ values of three biological replicates run in triplicate ± S.D. Means were determined to be different from 0 using two-way ANOVA with two-stage linear step-up procedure of Benjamini, Krieger and Yekutieli for multiple comparisons correction, * *p* < 0.05; ns = not significant (*p* = 0.0645).

In *C. parvum*, expression of the DNA meiotic recombinase 1 gene (*DMC1*) serves as a marker of transition into sexual stages [11]. MMV665917 inhibits expression of *DMC1*, thus inhibiting macrogamete development [11,26]. Since both trtE and MMV665917 were effective between 32 and 40 hpi (Figure 5) it, is possible that trtE is also inhibiting transition to the sexual stages, as MMV665917 has been shown to do [11]. To test this hypothesis, *C. parvum* infected cells were treated with either DMSO (vehicle control), trtE or MMV665917 for 4 h at either 40 or 44 hpi. After the 4 h of treatment, RNA was isolated for RT-qPCR of sexual stage gene expression markers. Percent inhibition of gene expression was calculated relative to DMSO control. Relative expression profile of sexual marker genes revealed that trtE significantly decreases *DMC1* expression when added at 40 hpi, compared to DMSO (Figure 6a). MMV665917 similarly reduces *DMC1* gene expression, corroborating previous findings [11]. Gamete specific gene markers *HAP2* (male) and *COWP4* (female) are expressed in concert with *DMC1* [11,13]. Data shows that trtE treatment reduces expression of *HAP2*, but not *COWP4,* when added at 40 hpi (Figure 6b,c). However, neither compound significantly affects the sexual gene markers when added at 44 hpi. These data suggests that trtE can inhibit *C. parvum* early in sexual development but does not affect the parasite once it has committed to sexual stages.

## 4. Discussion

*Cryptosporidium* parasites are a major public health concern due to their ubiquity, their capability to easily infect children, to cause chronic ailments in adults and severe, unresolvable symptoms in the immunocompromised population. Current treatments for cryptosporidiosis are poorly efficacious, thus new therapeutic alternatives are desperately needed. Top candidates with bioactivity against *Cryptosporidium* at sub micromolar concentrations and selectivity for parasite molecular targets inhibit calcium-dependent protein kinases [27,28] and tRNA-synthetases [29,30]. These compounds were discovered using target-based or phenotypic-based approaches [31] and are either in preclinical trials or late-lead discovery phase [32,33,34]. Libraries of approved compounds with activity against other pathogens have been screened to identify and repurpose drugs with activity against *Cryptosporidium* [35,36], thus accelerating clinical trials and approval processes. For example, clofazimine, an approved therapy for leprosy, was identified as an anti-cryptosporidiosis candidate through a drug repurposing screen [37]. While clofazimine had in vivo and in vitro efficacy against the parasite [37], the compound failed clinical trials because it did not reduce disease symptoms in HIV infected adults [38,39]. With so few drugs in the pipeline, characterization of new therapeutics with anti-cryptosporidial activity is a critical need.

TrtE is a macrodiolide polyketide synthesized by *Teredinibacter turnerae*, a symbiotic bacterium of shipworms [16] that inhibits the growth of multiple apicomplexan parasites at nanomolar to picomolar levels [17]. Multiple phenotypic based assays have been developed to pinpoint the stage at which compounds target *Cryptosporidium* and to narrow the search for the drug’s molecular target [11,31]. Using these strategies, we found that trtE inhibits 99.0% growth of *C. parvum* within an hour (Figure 2), prevents establishment of infection (Figure 3) and is most effective during the asexual stages of parasite development (Figure 5). Given that trtE kills *T. gondii* rapidly after two hours without discriminating between extracellular tachyzoite stages and parasites within the parasitophorous vacuole [17], it is sensible to infer that trtE is acting on the extracellular sporozoites. However, there is no difference in sporozoite viability when treated with either trtE or DMSO in suspension (Figure 4). Taken together, these observations suggest that trtE is inhibiting establishment of infection or targeting the parasite once it is inside the host cell.

MMV665917 and trtE are highly inhibitory between 32 and 40 hpi and exhibit a decline in efficacy if added at later timepoints (Figure 4). MMV665917 is a piperazine from the Medicines for Malaria Venture known to inhibit the transition to sexual stages and the macrogamete development of *Cryptosporidium* [11,26,33]. Both compounds were tested in parallel to evaluate the transition into the sexual stages. Interestingly, trtE inhibits *DMC1* expression similarly to MMV665917 (Figure 5a), suggesting that the compounds affect the transition to sexual stages when added prior to 40 hpi but do not affect those that have already committed after 44 hpi. Furthermore, trtE significantly reduces the expression of *HAP2* but not *COWP4* at 40–44 hpi, suggesting that the compound is targeting male gamete differentiation. Alternatively, it can be hypothesized that trtE targets actively replicating parasites and the decrease in HAP2 expression is a result of inhibition of microgamete replication. Further studies focused on the effect of trtE on sexual stages are needed to pinpoint the stage specificity of the compound.

TrtE is a unique compound that acts against multiple apicomplexan species at nanomolar concentrations [17]. Since forward genetic methods for trtE target identification failed, an RNAseq approach was tried to identify the target of this broad-spectrum compound. Transcriptomic analysis of *T. gondii* in the presence of trtE revealed the upregulation of a conserved coccidian gene, named tartrolon resistant gene (*trg*) [18]. However, *T. gondii* parasites lacking *trg* were only marginally more resistant to trtE, suggesting that *trg* was not the primary target of the compound. Furthermore, *Cryptosporidium*, and the hemoparasites also targeted by trtE [17], lack a *trg* homolog. For now, the target of trtE remains unknown.

While most natural products in drug discovery history have been isolated or derived from terrestrial sources [40], there has been a recent shift of focus to mining marine sources to uncover novel bioactive compounds for therapeutic use. Marine bioactive material (commonly isolated from bacteria, fungi, algae, sponges, and corals) has been shown to act against multiple eukaryotic pathogens [41]. For example, Leiodolide A, isolated from hard sponges, was discovered to have anti-*Cryptosporidium* activity [20]. Marinopyyrole A and its analogs, isolated from marine *Streptomyces*, have been shown to inhibit *T. gondii* [42]. Additionally, a multitude of marine alkaloids and their synthetic derivatives have been found to have activity against malaria and kinetoplastids [43]. These reports highlight the tremendous potential that marine-sourced compounds, like trtE, have as anti-parasitics.

## 5. Conclusions

Cryptosporidiosis is a growing public health concern worldwide. The marine natural product, trtE, inhibits *Cryptosporidium* growth in vitro and in vivo. TrtE rapidly kills asexual stage parasites and prevents establishment of infection possibly through inhibition of attachment and invasion or by preventing development of the early trophozoite. TrtE also inhibits the parasite during transition to sexual development and may also target male gametes. Further characterization of trtE is required to determine the molecular target of trtE.

## Figures and Tables

**Table 1:** EC_50_ of trtE against Nluc and WT *C. parvum* when measured by luminescence or qPCR.

*C. parvum* Strain	Quantification Method	EC50 (nM)	95% Confidence Interval (CI)
Nluc	Luminescence	15.9	13.8 to 18.4
Nluc	qPCR	14.8	10.4 to 21.7
WT	qPCR	11.2	7.6 to 16.3

## Data Availability

The data presented in this study are available on request from the corresponding author.

## Supplementary Materials

The following supporting information can be downloaded at: https://www.mdpi.com/article/10.3390/microorganisms10112260/s1. Figure S1: NMR spectra of purified trtE used in these experiements; Figure S2: TrtE does not affect *Cryptosporidium* excystation; Figure S3: Drug-response curve of MMV665917 against *C. parvum*; Figure S4: Time it takes MMV665917 to inhibit *C. parvum;* Figure S5: MMV665917 does not inhibit establishment of *C. parvum* infection.
