# Supplementary material for: The Marine Compound Tartrolon E Targets the Asexual and Early Sexual Stages of Cryptosporidium parvum
Source: Microorganisms. 2022 Nov 15;10(11):2260. doi: 10.3390/microorganisms10112260 (PMC9693555; doi:10.3390/microorganisms10112260)
Supplement: Supplementary file 1 [file microorganisms-10-02260-s001.zip › Supplemental figures.pdf]

Supplementary Figures

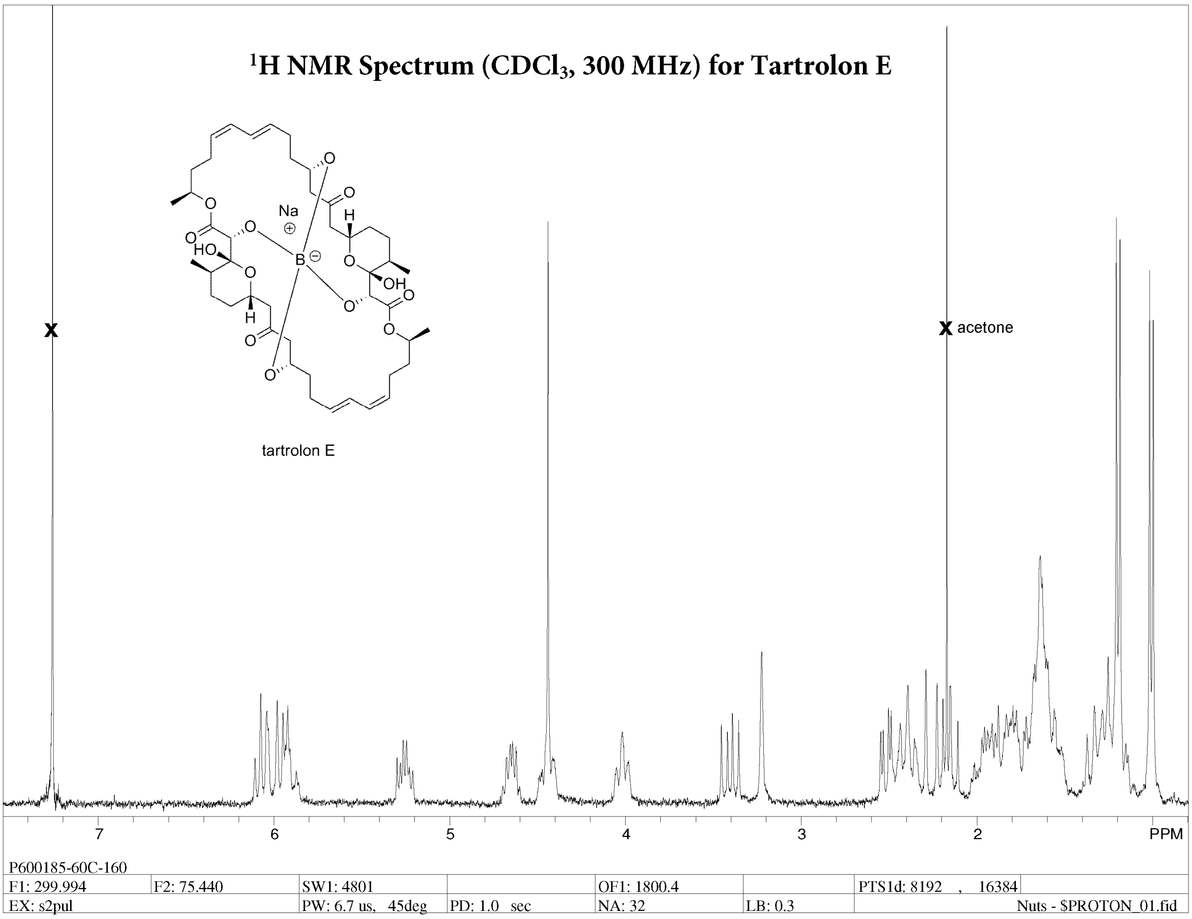

Figure S1: NMR spectra of purified trtE used in these experiements.

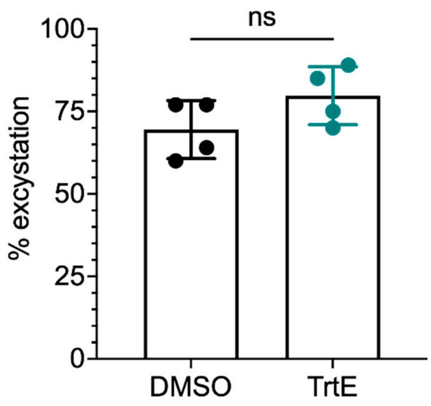

Figure S2. TrtE does not affect *Cryptosporidium* excystation. *C. parvum* oocysts were excysted for 2 hours in 0.6% taurocholate acid media in the presence of either DMSO or 100 nM trtE. The graph shows the average percent excystation ± SD of four independent experiments. Statistical significance was determined by an unpaired t test (p=0.1503), ns = not significant.

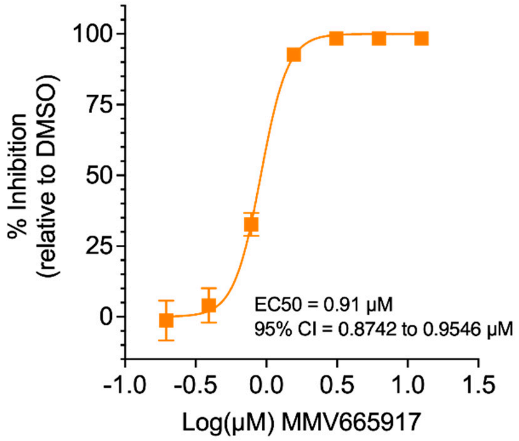

Figure S3: Drug-response curve of MMV556917 against *C. parvum*. HCT-8 cells were infected with *C. parvum*. Two-fold serial dilutions of MMV665917 were added to the parasites at 24 hpi. DMSO control was run in parallel. Parasite growth was measured at 72 hpi. The data plotted are the means ± SD of three independent experiments.

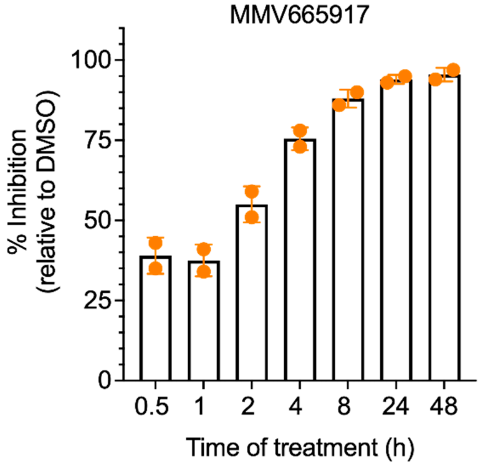

Figure S4: MMV665917 completely inhibits *C. parvum* growth after 8 hours**.** Parasites were treated with 6 μM MMV665917 at 24 hpi for 0.5, 1, 2, 4, 8, 24 and 48 hours with a DMSO treated control run in parallel. Parasite growth was measured at 72 hpi. Graph represents the percent inhibition of MMV665917 relative to DMSO control. Data plotted are the means ± SD of two independent experiments.

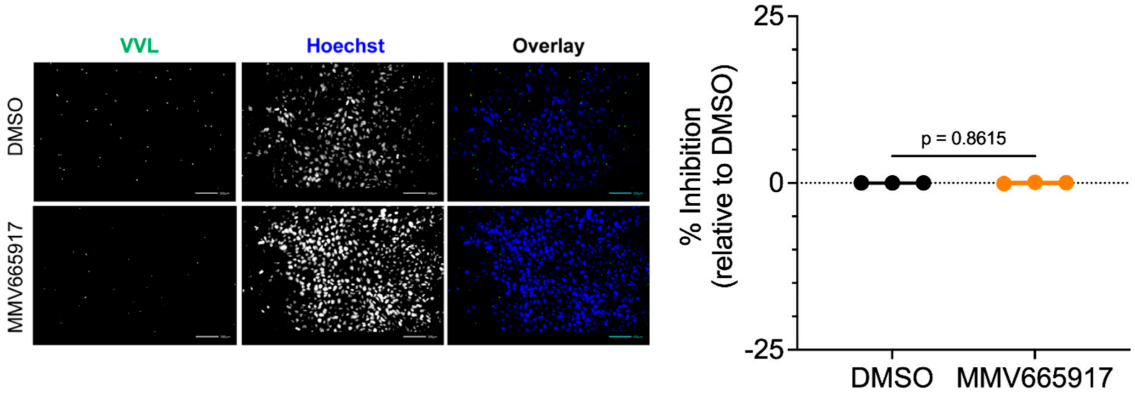

(**a**) (**b**)

Figure S5: MMV665917 does not inhibit establishment of *C. parvum* infection. Parasites were allowed to infect for 3 hours in the presence of 0.2% DMSO or 6 μM MMV665917. (**a**) Representative images of *C. parvum* infection. Parasitophorous vacuoles were stained with 1.33 μg/mL VVL and host cell nucleus with 0.09 mM Hoechst. Scale bar represents 50 μm. (**b**) Quantification of parasite invasion. Graphs shows percent inhibition of invasion with means ± SD of three independent experiments. Statistical significance was determined using an unpaired t test.
